# Three-minute critical appraisal of a case series article

**DOI:** 10.4103/0019-5413.77126

**Published:** 2011

**Authors:** Kevin Chan, Mohit Bhandari

**Affiliations:** McMaster University, Hamilton, Canada

Case series represents an observational study that reports on data from a subject group without a comparison population.^1^ In the hierarchy of evidence, it represents level IV evidence.^2^,^3^ This is due to lack of control subjects, making case series prone to bias. However, there is a substantial proportion of clinical studies that are case series in the medical literature,^2^,^3^ emphasising the importance of understanding its advantages and disadvantages. The following article explores the role of case series and examines the features of a well-designed study.

## KEY CRITERIA FOR CRITICAL APPRAISAL

Case series are often used to describe outcomes of novel treatments. The information gained can be used to generate hypotheses that lead to focussed studies of a stronger design.^2^ They are also helpful in refining new techniques or treatment protocols before they are studied in more advanced trials.^2^ The biggest advantage is that case series are feasible study designs, are easy to conduct and require less time and financial resources than randomised-controlled trials, case-control, or cohort studies.^2^ However, readers need to be conscientious of the inherent biases in case series, particularly selection bias. A poorly designed case series can have severe limitations in their conclusions. There are several key criteria that constitute a high-quality case series [Table 1].

**Table 1:** The three-minute checklist

Clear study objective/question
Well-defined study protocol
Explicit inclusion and exclusion criteria for study participants
Specified time interval for patient recruitment
Consecutive patient enrollment
Clinically relevant outcomes
Prospective outcome data collection
High follow-up rate

First, the study should have clear objectives with a well-defined, a priori study protocol.^2^ There should be consecutive patient enrollment and the inclusion and exclusion criteria should be explicitly stated.^2^ The time horizon or the duration of participant selection (including follow up period) should be stated up front as well. These study characteristics will help limit selection bias. Second, the outcome measures should be clinically relevant and collected prospectively.^2^ Ideally, the use of health-related quality of life measures will help strengthen the value of the case series. Finally, a high follow-up rate is imperative.^2^ If a large number of patients are dropping out of a study, the validity of the treatment or study protocol must be questioned. Unfortunately, even the best designed clinical trials can lose patients to follow-up. In such situations, it is important that the authors state the reasons for the loss to follow-up.

The study by Bravo^3^ provides a good illustration of a well-designed case series. This study looked at the outcomes of proximal interphalangeal joint arthroplasty using a relatively new prosthesis called pyrolytic carbon implants. The authors attempted to limit bias by stating explicit details about surgical indications and contraindications for arthroplasty, as well as the surgical technique and postoperative therapy employed for all study participants. They enrolled consecutive patients and clearly specified the duration of recruitment of participants. Furthermore, the authors examined important patient outcomes such as range of motion, pain, satisfaction, and grip strength. To be critical though, the use of a validated, quality of life measurement tool would be more ideal, such as the Disabilities of the Arm, Shoulder, and Hand questionnaire. Finally, there were essentially no patients loss to follow-up, with the exception of one, who unfortunately died during the study period.

## SUMMARY

In conclusion, a case series can be prone to bias, limiting its generalisability to larger populations of patients. However, a well-designed case series can provide information that allows hypotheses to develop, leading to further advanced studies. It is important for readers to recognise the advantages and disadvantages of case series.
